# Evaluating the Impact of a Daylight-Simulating Luminaire on Mood, Agitation, Rest-Activity Patterns, and Social Well-Being Parameters in a Care Home for People With Dementia: Cohort Study

**DOI:** 10.2196/56951

**Published:** 2024-11-29

**Authors:** Kate Turley, Joseph Rafferty, Raymond Bond, Maurice Mulvenna, Assumpta Ryan, Lloyd Crawford

**Affiliations:** 1 School of Computing, Ulster University, Cromore Rd, Belfast, BT52 1SA, United Kingdom, 44 28 7012 3456; 2 School of Nursing and Pandemic Science, Ulster University, Belfast, United Kingdom; 3 Chroma Lighting, Belfast, United Kingdom

**Keywords:** digital health, dementia, dynamic lighting, sensors, circadian rhythm, daylight, wellbeing, mood, agitation, sleep, social wellbeing, care home, older adults, elderly, cardiac, psychological, monitoring

## Abstract

**Background:**

Living with a diagnosis of dementia can involve managing certain behavioral and psychological symptoms. Alongside cognitive decline, this cohort expresses a suppression in melatonin production which can negatively influence their alignment of sleep or wake timings with the 24 hour day and night cycle. As a result, their circadian rhythms become disrupted. Since daylight has the capacity to stimulate the circadian rhythm and humans spend approximately 90% of their time indoors, research has shifted toward the use of indoor lighting to achieve this same effect. This type of lighting is programmed in a daylight-simulating manner; mimicking the spectral changes of the sun throughout the day. As such, this paper focuses on the use of a dynamic lighting and sensing technology used to support the circadian rhythm, behavioral and psychological symptoms, and well-being of people living with dementia.

**Objective:**

This study aimed to understand how dynamic lighting, as opposed to static lighting, may impact the well-being of those who are living with dementia.

**Methods:**

An ethically approved trial was conducted within a care home for people with dementia. Data were collected in both quantitative and qualitative formats using environmentally deployed radar sensing technology and the validated QUALIDEM (Quality of Life for People With Dementia) well-being scale, respectively. An initial 4 weeks of static baseline lighting was used before switching out for 12 weeks of dynamic lighting. Metrics were collected for 11 participants on mood, social interactions, agitation, sense of feeling, and sleep and rest-activity over a period of 16 weeks.

**Results:**

Dynamic lighting showed significant improvement with a moderate effect size in well-being parameters including positive affect (*P*=.03), social isolation (*P*=.048), and feeling at home (*P*=.047) after 5‐10 weeks of dynamic lighting exposure. The results also highlight statistically significant improvements in rest-activity–related parameters of interdaily stability (*P*<.001), intradaily variation (*P<*.001), and relative amplitude (*P*=.03) from baseline to weeks 5‐10, with the effect propagating for interdaily stability at weeks 10‐16 as well (*P<*.001). Nonsignificant improvements are also noted for sleep metrics with a small effect size; however, the affect in agitation does not reflect this improvement.

**Conclusions:**

Dynamic lighting has the potential to support well-being in dementia, with seemingly stronger influence in earlier weeks where the dynamic lighting initially follows the static lighting contrast, before proceeding to aggregate as marginal gains over time. Future longitudinal studies are recommended to assess the additional impact that varying daylight availability throughout the year may have on the measured parameters.

## Introduction

Experiencing life with an atypical body clock can often materialize as a result of living with dementia. In fact, studies have shown that the relationship between atypical body clocks and dementia may even be bidirectional [1]. This means that the disruption of one’s body clock may catalyze their progression of dementia and vice versa. The combined effect of this can lead to disjointed rest-activity patterns and poor sleep quality, which then impacts mood and well-being in the longer term [2,3].

At present, there are over 55 million people living with dementia around the world [4]. This is 55 million people living without a cure for their diagnosis. As a result of this, society places great importance on research which can either predict the diagnosis in advance or alleviate the symptoms after the fact in a preventative versus reactionary approach [5].

This paper focuses on the latter approach, exploring means to alleviate symptoms of dementia to improve well-being. To expand the above content, a disrupted body clock (aka circadian rhythm) is a common factor of natural ageing, and is heightened in people living with dementia [6]. This disruption lends itself to poor sleep quality. In turn, this misaligns the typical timing of one’s sleep and wake cycle with the timing of sunset and sunrise which acts as our inherent, evolutionary alarm clock. Alongside this 24 hour cycle of light and dark, our bodies experience a diurnal cycle of the hormones melatonin and cortisol [7]. Melatonin is a hormone which encourages relaxation and sleep while cortisol is commonly named the “stress hormone” and governs the alerting response in the body. In a typical body clock aligned with sunlight cues, melatonin levels will begin to rise in the evening hours at the onset of darkness, and cortisol levels peak in the afternoon when sunlight is wholly available [8]. With dementia, the disrupted body clock suppresses this cycle of melatonin to cortisol and there becomes a discrepancy between the signals within the body, which should control our sleep-wake rhythm at these predefined times of the day. When sleep and hormone cycles are not in synchronization, the interlocking of these mechanisms becomes chaotic and thus a disrupted circadian rhythm and its symptoms ensue [8]. Since the circadian rhythm is responsible for controlling mood, body temperature, appetite, sleep-wake cycles, rest-activity levels among other factors, ensuring its synchronization is essential for supporting well-being for people living with dementia.

Objectively, it is known that light is the strongest zeitgeber (german for time-giver) for the human body and its circadian rhythm [9]. The circadian rhythm is governed from the suprachiasmatic nucleus in the brain, which is accessed via pathways from the eye. Within the eye, there are rods and cones which receive lighting spectra and translate this into a visual response. However, the discovery of the intrinsically photosensitive retinal ganglion cells (ipRGCs) in 2002 supported the theory that light delivers both photopic and melanopic contributions within human systems [10]. In other words, light is not only necessary for visual purposes (photopic) but also for aligning the circadian rhythm (melanopic). These ipRGCs exhibit varying sensitivity to different wavelengths of light, peaking at 480 nanometers (blue colored light) [10]. This means that the incorporation of dynamic lighting as opposed to static lighting may be critical for attuning the circadian rhythm with the analogous varying properties of daylight with which we have evolved.

Although, however inherent the need for lighting is to align the human circadian rhythm, the extent to which this applies in dementia is largely uncertain [11,12]. Typically, this is because the nature of the disease varies largely on factors such as the state of progression of dementia, alongside different factors that affect the response to lighting: chronotype, age, gender, and previous lighting exposures [13]. Additionally, the methods for obtaining the amount of lighting one is exposed to are commonly not accounted for, and the units of the lighting spectra trialled are far from systematic [14]. This makes comparison of lighting trials and studies within dementia cohorts difficult, resulting in a critical gap in knowledge within this field. This study aims to address some of these gaps by monitoring aspects of well-being and correlating this with commonly reported lighting metrics.

In order to monitor the above, it is critical to collect information on the daily activity and well-being of people living with dementia [15]. As such, this study makes use of integrated, environmentally deployed sensors to enrich this dataset in a nonintrusive manner. This setup allows for the formation of a technology, which can deliver circadian-aligned lighting and simultaneously monitor any resultant changes to well-being. Additionally, validated questionnaires are used to collect information on certain behavioral symptoms, which the sensors cannot themselves generate. Collaboratively, this information will provide informatics in response to dynamic lighting systems for people living with dementia. This study therefore hypothesizes that all measured parameters of well-being will benefit from exposure to dynamic lighting as opposed to static lighting over time.

## Methods

### Study Design

A combined lighting and sensing intervention was carried out from March to July 2023 for a period of 16 weeks, whereby participants with dementia experienced a change in their received lighting output. The technology is designed to deliver dynamic lighting to support the circadian rhythm of people living with dementia.

Simultaneously, it is designed to monitor the change to certain aspects of their circadian rhythm and well-being that are expected to be impacted by changes in the lighting spectra. The trial invoked a baseline-intervention strategy on the same group of individuals to obtain repeated measures at several key points throughout this study.

### Ethical Considerations

This study has been approved by the Office for Research Ethics Committees Northern Ireland under Integrated Research Application System ID 311547. Recruitment of participants was managed via the care home manager acting as gatekeeper. With consideration of the inclusion criteria, a dementia-friendly information leaflet was circulated by the care home manager to the residents and family members of people living with dementia. A similar leaflet was delivered to the care staff. Those who were interested in participating were invited to a presentation afternoon in the care home where 2 members of the research team brought the luminaire or sensor prototype to the care home and explained this study’s requirements for all involved parties.

Care staff were shown the questionnaire and user guide and offered to take part. After this, for those who were willing, initial consent was obtained and reaffirmed verbally throughout this study. In addition to care staff providing informed consent for their contribution to questionnaires, family members were asked to provide proxy consent on behalf of the selected residents recruited by the care home manager. For the residents living in this unit, all of their families possessed the legal right to consent to matters on their behalf that relate to their everyday life and well-being. This was communicated to the researchers by the care home manager. Since all participants had moderate-to-severe stages of progression of dementia and the fact that this was a pilot study requiring only passive interactions with the technology, it was deemed appropriate by family members, researchers, and the care home manager to gain initial consent via proxy at this stage of this study. A paper copy of a consent form was given to the participant’s proxy to be signed. If consent has been given, the participants data will be collected and analyzed by the research team. If at any point a participant proxy retracts their consent or the resident with dementia indicates they do not wish to be part of this study (verbally or otherwise), the data up until that point will have been collected and analyzed, and any data collected after this may be stored but not used for analysis, or exempt from collection altogether if requested. Proxies for participants were informed should they wish, to opt in and opt out at any point by informing the care manager, care staff, or chief investigator.

### Study Protocol

**Figure 1.**
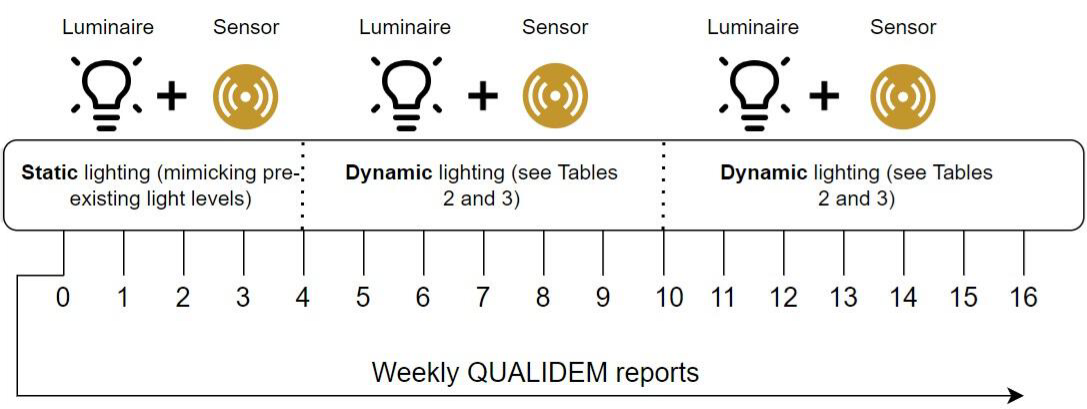
Baseline lighting was static in nature and was used as a control for 4 weeks. Dynamic lighting was introduced from weeks 5-16 inclusive. Sensor metrics were collected 24/7 for all 16 weeks with QUALIDEM reports completed on a weekly basis. QUALIDEM: Quality of Life for People With Dementia.

This study was implemented using the following protocol as seen in Figure 1.

- Weeks 1‐4 : baseline lighting and static lighting
- Weeks 5‐16: dynamic lighting

The baseline lighting was used as a control for 4 weeks to generate differences in well-being attributed to the dynamic lighting. After these 4 weeks, the lighting program was changed to exhibit the dynamic lighting spectra. The period of 12 weeks to trial the dynamic lighting was chosen due to the fact that it is estimated to take approximately 2‐5 weeks to realign a circadian rhythm, therefore providing ample opportunity to witness potential progression over time [16]. As such, a baseline (weeks 1‐4), midpoint (weeks 5‐10), and end of intervention (weeks 10‐16) approach for analysis was used on the results to observe the progression in well-being throughout the trial. Alongside the sensing metrics collected, the trial required insight into additional well-being parameters that could not be collected via the sensors. As such, a validated well-being questionnaire (QUALIDEM) was also completed by care staff on a weekly basis [17]. The QUALIDEM scale represents the “Quality of Life for People With Dementia” and is a validated measure of quality of life and well-being for people living with dementia [17]. It is used to understand the behavior and attitude of people living with dementia on a weekly basis. The scale measures 10 aspects of quality of life; (A) care relationship, (B) positive affect, (C) negative affect, (D) restless tense behavior, (E) positive self image, (F) social relationships, (G) social isolation, (H) feeling at home, (I) occupation, and (J) other questions for further research. For this study, the latter two parameters (I and J) are presented within the report but excluded from analysis as these are not factors anticipated to be impacted by the circadian rhythm in response to lighting. The QUALIDEM scale is to be completed by care staff on behalf of the residents with dementia. Although there may be unintentional bias on reporting scores, it was deemed suitable for multiple care staff to complete the QUALIDEM scales due to its high Inter Rater Reliability score [18]. This also facilitated the reduction in caregiver workload and burden during the week.

Each resident was free to enjoy their daily routines normally and no constraints on their activity was requested. The sole hypothesis tested in the trial was the changes to various aspects of well-being under exposure to static versus dynamic lighting spectra.

### Hardware and Architecture

**Figure 2.**
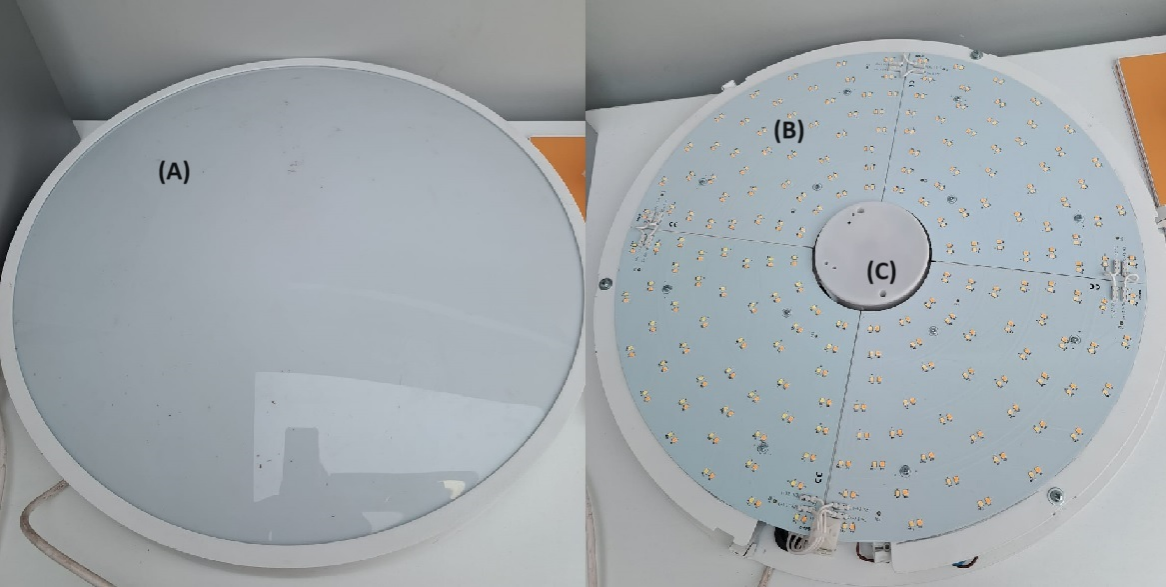
Lighting and sensing device. Device includes (A) diffuser, (B) tuneable white LED quadrants, and (C) radar sensor mounted on a base plate.

The bespoke device deployed within the care home can be seen in Figure 2. It has been designed to integrate both lighting and sensing components of the technology in a feedback setup. The lighting output is produced by the dimmable, tuneable-white LED boards which can change in intensity and color temperature. The integrated sensor is a radar sensor, which can track movements on a frame-by-frame basis at fine-grained resolution. The sensor and luminaire have bidirectional communication pathways with a cloud storage, enabling data-driven feedback to inform the lighting. This means that the luminaire is bespoke in the fact that it has the capacity to actuate changes in the lighting spectra based on data-driven insights. For example, if a resident is observed to frequent their bed during the day for a nap, it could be possible to create a more “alerting” lighting environment (more blue output at higher intensities) to help aid their wakefulness in the day and resultantly promote their sleep during the night. However it should be noted that this pilot study is comparing a predetermined dynamic spectra (consistent with the daylight spectra) to a static lighting spectra in order to generate an initial foundation for how lighting impacts well-being for people living with dementia in order to ensure that the future data-driven actuation will be suitable for each individual.

**Figure 3.**
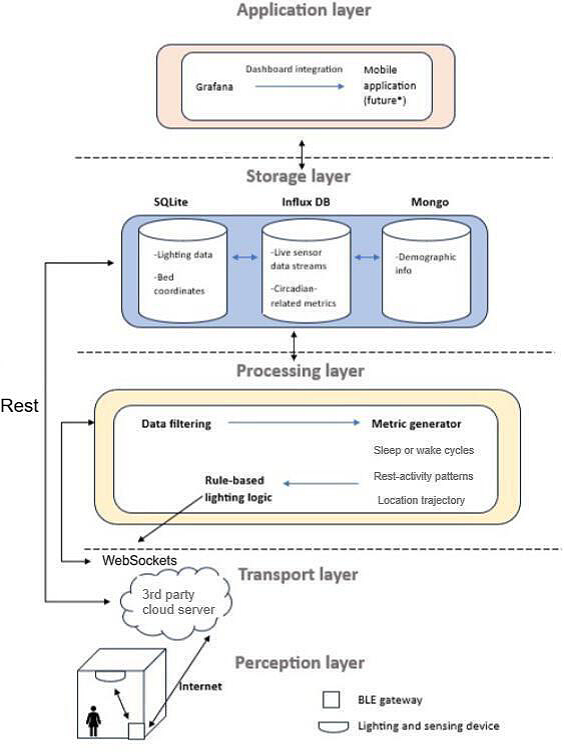
IoT architecture of smart care environment. Data is transferred across the network to storage and accessed by algorithms performing metric generation for visualization. BLE: Bluetooth low energy; DB: database; IoT: internet of Things.

The device communicates via Bluetooth protocol to the next device or node in the network, creating a mesh network of the lighting and sensing devices. This mesh then interacts with an edge node operating as a Bluetooth low energy gateway and transferring data to a 3rd party cloud platform. From here, the data is accessed via WebSockets (IETF) and consumed by the backend logic for processing and visualization. The entire architecture can be seen in Figure 3.

The data collected include both lighting and sensor data harvested from the network, in 15 minute intervals and real time, respectively. Due to the range of Bluetooth protocol, several repeater nodes had to be included at regular distances to bridge the communication between devices. Figure 3 demonstrates the Internet of Things architecture for the home implementation. The real time sensor data is filtered and consumed by the metric generator. This generator invokes algorithms which search for the activity levels for the previous 15-minute interval alongside the sleep-wake metrics for each day and night (see further detail in the Sensing Component section). In addition, the live location is also assessed and reported to the influx database for access on the visualization platform.

### Site

**Figure 4.**
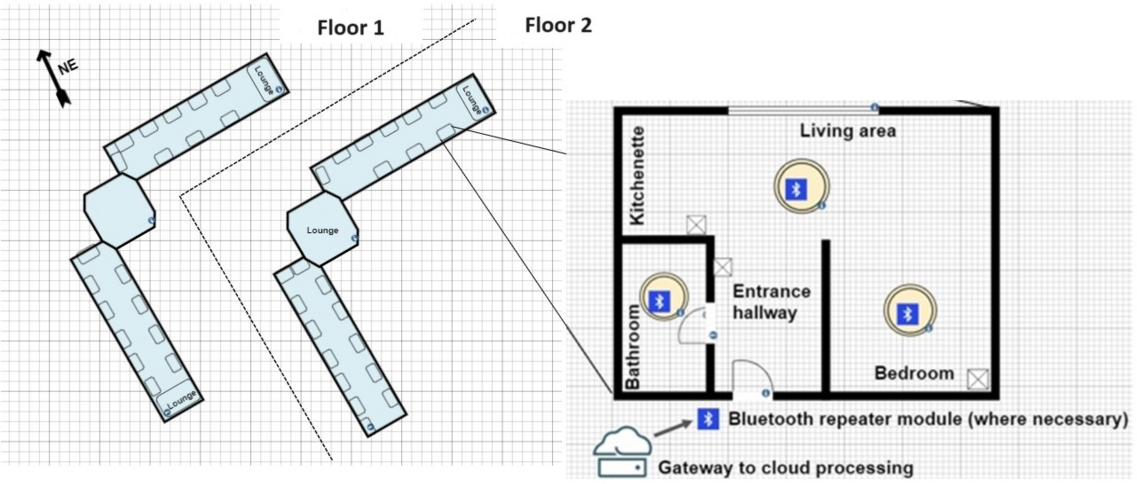
Floor plan of the care home. Individual flats contained a kitchenette, living area, bathroom and bedroom with access to communal lounges or common areas in each wing. Further, 3 lighting and sensing devices were placed in each flat. NE: northeast.

The technology was trialled in a care home within the United Kingdom. The site accommodates up to 42 residents with a dedicated wing for dementia care. Various rooms were included in this study between the upper and lower corridors, totaling 11 rooms. Five rooms faced northeast, 3 rooms faced southwest, 2 rooms faced northwest, and 1 room faced southeast. Each room is an independent living quarter for every person with dementia, with a shared common area for each wing where one can dine and watch television, as shown in Figure 4. All rooms experienced a similar deployment, with a device in each of the kitchenette and living area, bedroom, and bathrooms. The common areas were also equipped with several dynamic lighting devices but with the sensors removed to compensate for those present within these areas who had not consented to data collection.

### Participants

Eleven residents participated in the intervention as seen in Table 1; all with a diagnosis of dementia. Each individual’s type of dementia and Mini-Mental State Examination was not logged, however all participants were known to exhibit a moderate-severe state of progression. Participants were included based on the criteria that they reside at the care home and had a diagnosis of dementia. Participants were excluded on the basis that they experience schizophrenia, epilepsy, restless leg syndrome, serious eye disease such as retinitis pigmentosa or cataracts or blindness, mania, historical head injuries, diabetes, or any physical disability which would restrict the participant to their bed. These criteria were assessed by the care home manager and relevant participants selected for recruitment. Upon recruitment, 13 participants were identified and consented to this study. Eleven participants were included in the final trial due to the layout of the building and restriction in the Bluetooth range between large distances with concrete walls between. During this study, participants could live their lives as they would prior to the lighting trial. The care home has a scheduled time for breakfast, lunch, and dinner with the option to have a staggered start to accommodate all residents; aside from this, residents are given autonomy to settle in any area of the care home wing including a connected outdoor or indoor walkway, their flatlets, common areas, or their neighbor’s flatlets. At night, residents are encouraged by care staff back to their flatlets and into bed and are checked on in 2 hourly intervals.

**Table 1.** Participant details.

Variable		Details
Participants, n		11
Female, n		11
Male, n		0
Age (years), mean (SD)		87.45 (2.05)
Weight (kg), mean (SD)		57.45 (1.72)
**Medications, n**		
	Diazepam (anxiety)	2
	Memantine (dementia)	9
	Risperidone (antipsychotic)	6
	Donepezil (dementia)	4
	Circadin (sleep)	1
	Sertraline (antidepressant)	4
	Mirtazapine (depression and anxiety)	1
	Melatonin (sleep)	1
	Citalopram (low mood)	1

### Lighting Component

First, the dynamic lighting was designed to align with the photopic lighting requirements known for the ageing population from existing lighting guidance [19]. These are typically reported as illuminance at eye level measured in lux alongside measurements for the correlated color temperature of the lighting reported in Kelvin. Second, the lighting was required to be of significant spectra to ensure a melanopic response would be invoked [14,20,21]. This response is most recently reported in terms of either circadian stimulus (CS) or the *α*-opic Melanopic Equivalent Daylight Illuminance (M-EDI) lux [20,21]. The former is defined as the “effectiveness of the spectrally weighted irradiance at the cornea from threshold (CS=0.1) to saturation (CS=0.7)” [20]. The latter refers to International System of Units–compliant metrics as supported by the International Commission on Illumination, which converts multiple spectral inputs to ipRGC-relevant quantities [21]. We have used this to obtain additional values of M-EDI lux according to the LED spectral distribution and melanopic ratio of the lighting. The LED board is bespoke and has been designed to optimize the melanopic response.

In this trial, the illuminance was measured using a lux meter and the color temperature was obtained from the luminaire network. The calculations for the melanopic metrics were completed using the spectral power distribution values from our LED datasheet [22] and the lux intensities from Table 2, with the aid of open access 3rd party software for both CS and M-EDI lux calculations [20,23]. The melanopic ratio does not change throughout this study. The previously installed static lighting for the care home’s bathrooms, bedrooms, kitchennette and living space, and common areas exhibit mean illuminance of 99 (SD 49), 75 (SD 12), 59.33 (SD 15.41), and 147 (SD 60.42) lux, respectively. In contrast, the dynamic intervention metrics are shown in both Tables2  3.

**Figure 5.**
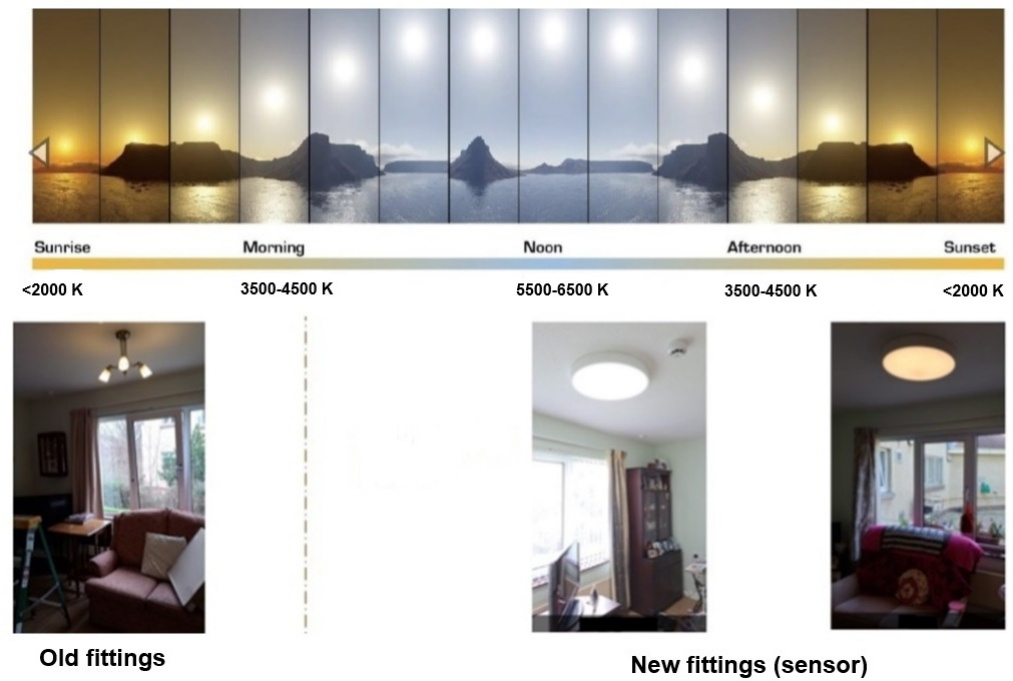
Lighting before and after intervention highlighting the variation in spectra throughout the day.

The intervention lighting was programmed to simulate the varying properties of the sunlight cycle throughout the day as depicted in Figure 5. Warmer color temperatures were present in the early mornings and evenings with cooler color temperature in the middle of the day. The capacity to switch on or off the lighting was maintained by the residents or the care staff according to preference and was also logged in the network. This then allows for metrics on actual lighting exposures to be calculated.

**Table 2.** Photopic lighting intervention parameters.

Intensity (lux)	Correlated colour temperature (K)	Output (%)^a^	Time
300	3350	90	7 AM to 8 AM
550	4000	100	8 AM to 8:30 AM
550	5200	100	8:30 AM to 9 AM
550	6500	100	9 AM to 2:30 PM
300	4200	90	2:30 PM to 3 PM
260	3400	90	3 PM to 4 PM
300	2700	90	4 PM to 6 PM

a The output is from the luminaire manufacturer and is only expressed in percentages. The actual values are not available. The “intensity (lux)” column states what the authors can measure at eye level that reflects what the percentages give us at this amount of distance from the lighting.

**Table 3.** Melanopic lighting intervention parameters.

Intensity (lux)	Circadian stimulus	M-EDI^a^ lux
260	0.404	331
300	0.431	382
550	0.532	700

a M-EDI Melanopic Equivalent Daylight Illuminance.

### Sensing Component

**Figure 6.**
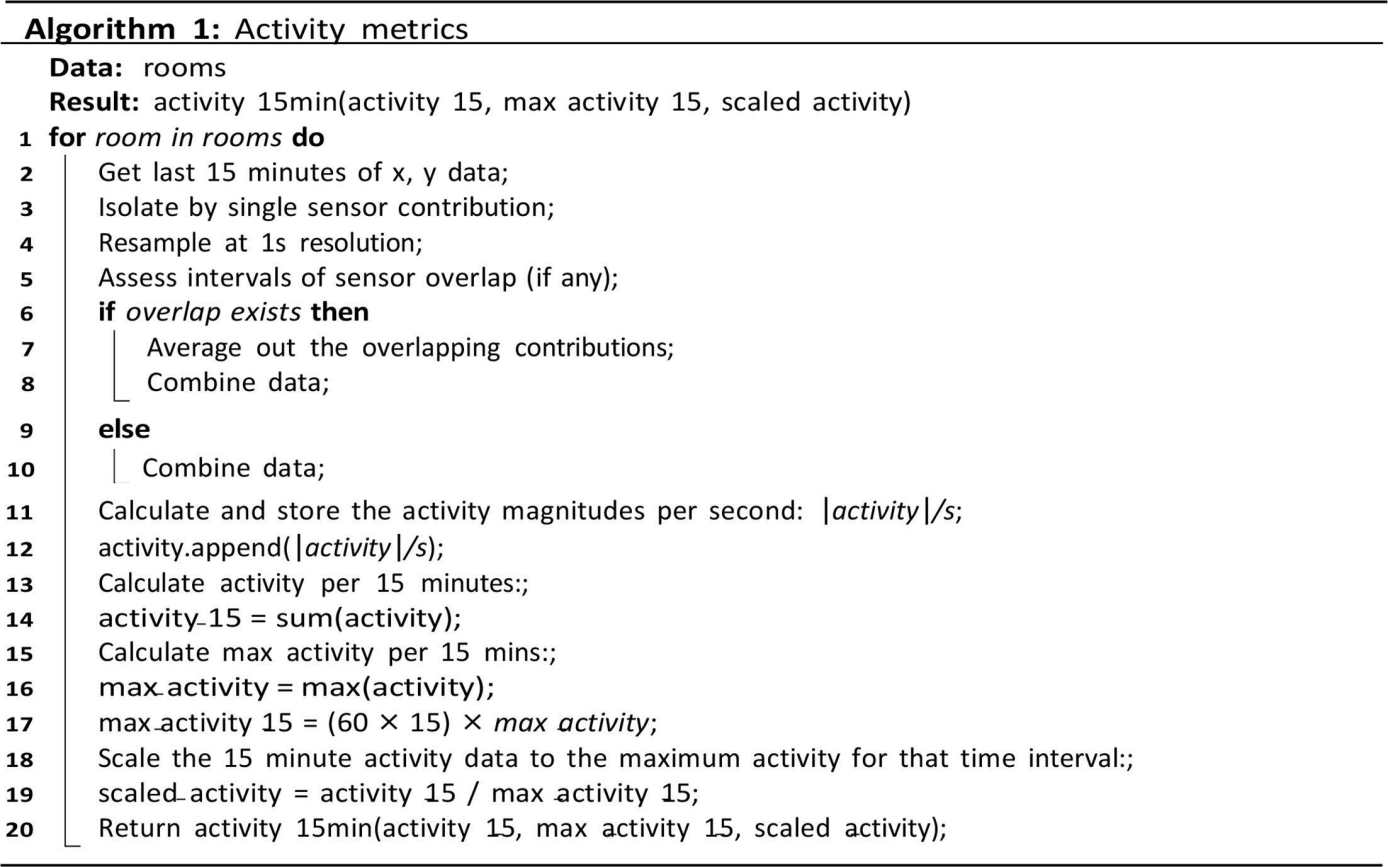
Algorithm 1: activity metrics.

The sensor is an environmental sensor integrated inside the luminaire and concealed by the diffuser. It is a depth sensing radar sensor, which can perceive the averaged 3D coordinates of an individual in a privacy-friendly manner. The sensor is deployed to track the location, rest-activity, and night activity metrics of the residents with dementia; all parameters expected to be influenced by changes to the circadian rhythm. Frame-by-frame x-y translations were captured at 8 Hz and the total magnitude calculated over 15-minute intervals. This was normalized by the maximum total translation in the current 15-minute window in order to give activity levels as a percentage. As such, the pseudocode below Figures6  7 demonstrates the type of information collected about each resident.

**Figure 7.**
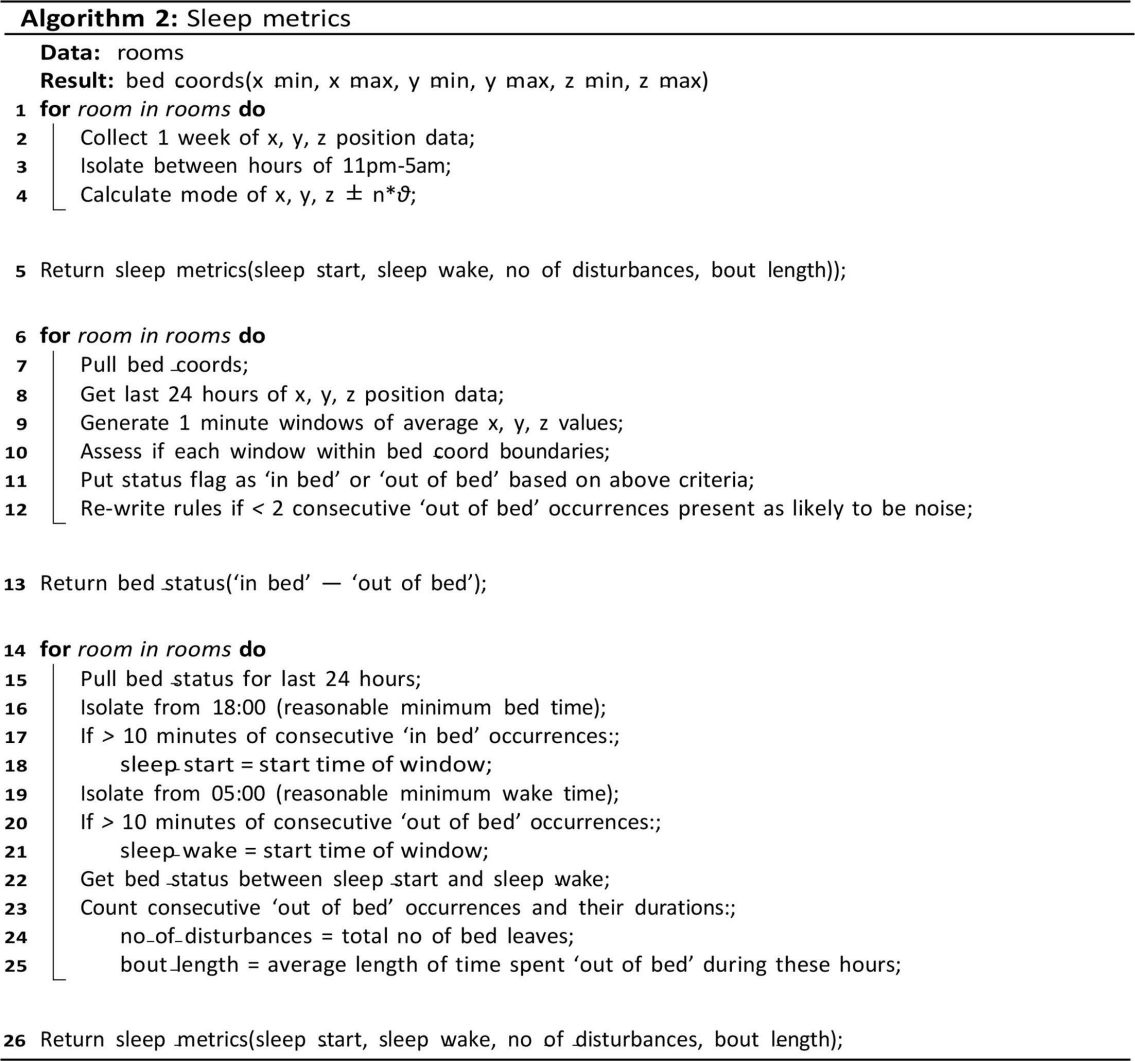
Algorithm 2: sleep metrics.

### Data Analysis

Data analyses was undertaken using SPSS software [24]. All datasets were tested for normality using the Shapiro-Wilk test since the cohort was less than 50 participants. If the data was parametric, paired samples *t* tests (2-tailed) were used to determine statistical significance. If the data exhibited a nonparametric distribution, the Wilcoxon-Signed rank test was used between related samples. Effect sizes were calculated using Cohen *d* and Cliff δ for parametric and nonparametric data, respectively. All measured parameters of well-being were determined parametric with significant deviation from normal and detected solely for 2 parameters of the QUALIDEM scale (E and H: positive self-image and feeling at home). These were therefore analyzed according to the nonparametric methodology highlighted above. Due to the heterogeneity in the sleep metrics obtained, the median of the datasets was calculated to allow for less skew that would arise from taking an average.

## Results

### Lighting

**Figure 8.**
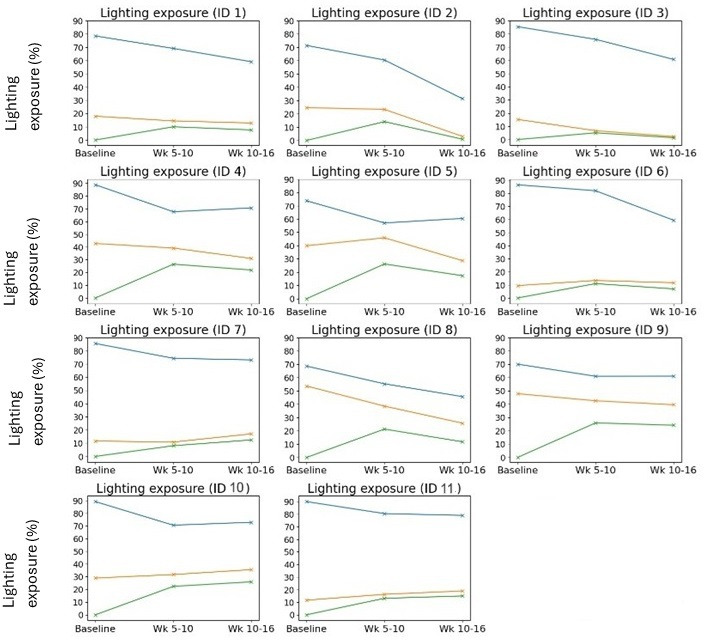
Lighting exposure per participant. Blue represents the percentage time in sensor areas, orange is the percentage time while in these areas with lighting switched on, and green represents the percentage time of exposure to dynamic lighting.

The lighting metrics were harvested from the network to estimate how much time a resident spent under the dynamic lighting intervention over the duration of the trial. This can be seen in Figure 8. The blue line represents the amount of time spent in their rooms. The orange line indicates that while in their rooms, this is the average percentage of time that the lights were switched on and they were in fact receiving the dynamic lighting intervention. Green lines represent the percentage exposure to dynamic lighting. Typically, the care staff note that common room lighting is generally switched on during the hours of 8 am to 8 pm and all resident lighting is switched on upon wake up time and altered at their discretion throughout the day.

**Figure 9.**
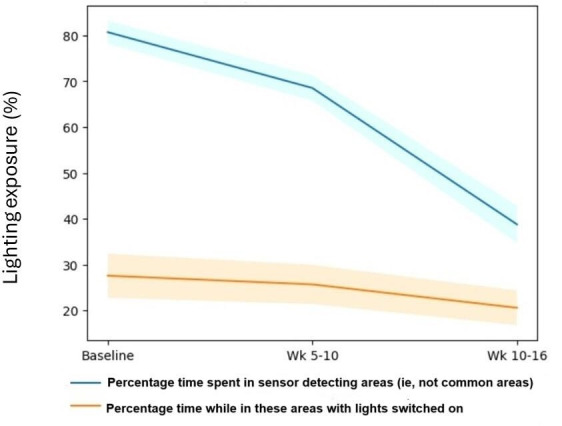
Time spent with lights on as a group trend. The lighter lines indicate error boundaries.

A group analysis of Figure 8 can be seen in Figure 9. The amount of time spent in their rooms generally decreased meaning the amount of time assumed spent in common areas increased. Although both the blue and orange lines are decreasing, they are converging.

Assuming the times not spent in their rooms were considered to be under common area fully dynamic lighting, the general percentage exposure to dynamic lighting from baseline to weeks 5‐10 to weeks 10‐16 increased from the following:

- Baseline: 0% exposure as fully static lighting
- Weeks 5‐10: 37.48% exposure (SD 2.77%)
- Weeks 10‐16: 53.08% exposure (SD 4.07%).

### QUALIDEM

**Figure 10.**
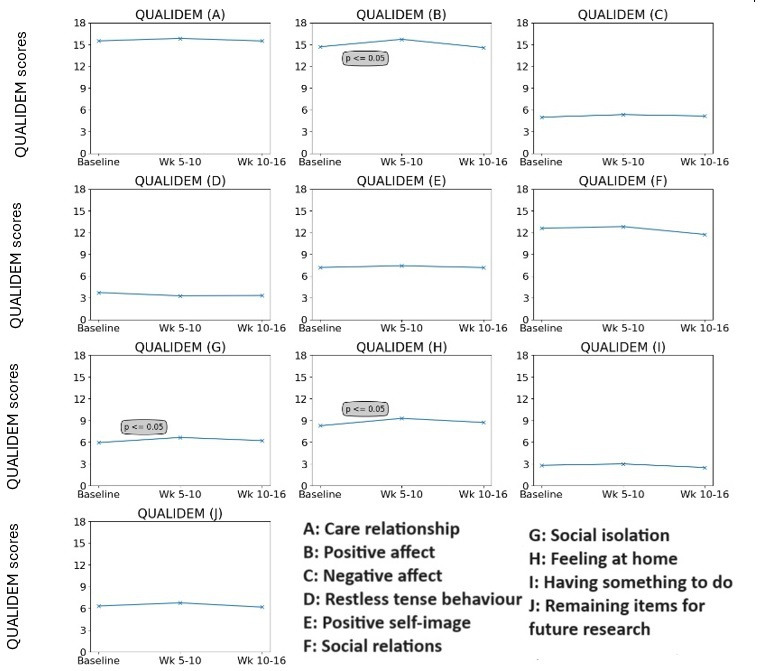
QUALIDEM scores as a group analyses during both baseline (static lighting) and weeks 5-10/10-16 (dynamic lighting). Note items I and J are not analyzed in this study. Larger values indicate improvement. QUALIDEM: Quality of Life for People with Dementia.

Figure 10 shows the QUALIDEM parameters analyzed independently in accordance with the documentation. The results show that from baseline to weeks 5‐10, there was a statistically significant improvement in B, G, and H with values of *P*=.03, *P*=.048, and *P*=.047 and a moderate effect size. This corresponds to statistically significant improvements in positive affect, social isolation, interdaily stability (IS), and feeling at home.

### Activity

Commonly reported metrics used in rest-activity studies are the outputs of IS, intradaily variation (IV), and relative amplitude (RA) [25]. IS is an indicator for the synchronization to light and other metabolic cues that inform the body clock. It takes a value between 0 and 1 with the larger number indicating better synchronization. IV gives an indication of body clock disruption and sleep efficacy, with possible values between 0 and 2. The lower the value the better sleep efficacy. RA gives an indication of the robustness of the daily rest-activity rhythm, with higher values equating to a more robust cycle [25].

**Figure 11.**
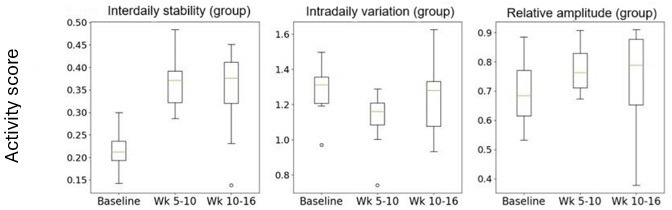
Box plots for rest-activity metrics as a group analysis, including interdaily stability, intradaily variation, and relative amplitude.

On a group level, the IS and IV both experience a significant improvement from baseline to weeks 5‐10 with values of *P*≤.001 for both variables (Figure 11). The IS also reflects this from baseline to weeks 10‐16 with a significance of *P≤*.001. The IV increases from baseline to weeks 10‐16 but not significantly with a *P* value of .22.

**Figure 12.**
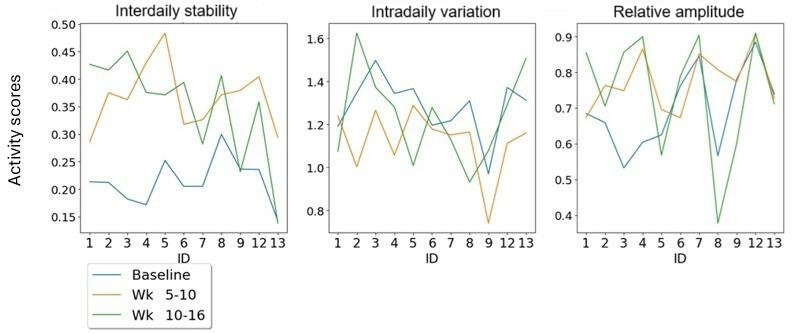
Line plots for rest-activity metrics for an individual analysis, including interdaily stability, intradaily variation, and relative amplitude.

Figure 12 shows the individual RA. From baseline to weeks 5‐10, there was a significant improvement in the RA, which means the participant experiences a more robust 24 hour rhythm. The *P* value for this period was .03. The baseline to weeks 10‐16 was not significant, but it follows a general upward trend on a group analysis.

**Figure 13.**
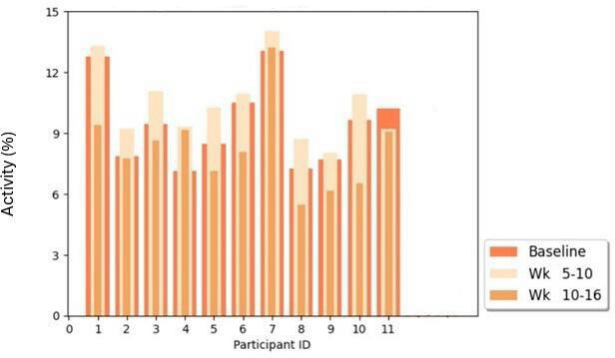
This is the percentage activity levels in the 4 hours before sunset during the trial under different lighting exposures. These data were analyzed with a view to compare with sunset activity or sundowning.

An assessment was also carried out of activity levels during typical sundowning hours. The 4 hours before sunset (8 PM, 9 PM, and 10 PM for baseline, weeks 5‐10, and weeks 10‐16, respectively) were analyzed as seen in Figure 13. The activity levels increased significantly from baseline to weeks 5‐10 (*P*≤.001) but then decreased significantly from baseline to weeks 10‐16 (*P*=.01). If we take the sunset activity as an analogy to activity during the sundowning hours in dementia, we can see that activity during sundowning hours seems to have increased at weeks 5‐10 and decreased at weeks 10‐16.

### Sleep

**Figure 14.**
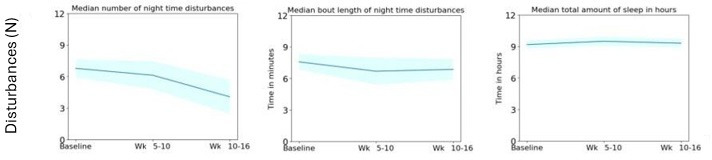
This is the sleep-related metrics recorded on a group level, highlighting the median number of night-time disturbances, bout length of these night-time disturbances, and the total amount of sleep in hours.

Figure 14 shows the median number of sleep disturbances as a group decreased from both baseline to weeks 5‐10 and from baseline to weeks 10‐16; however, this did not reach statistical significance.

It should be noted that the highest number of disturbance counts throughout the night on average happened to participant 5 who is the sole one on sleeping medication. If the paired *t* test is repeated for the group without participant 5’s data, the reduction in sleep disturbances becomes statistically significant from baseline to weeks 10‐16.

Figure 14 shows the median length of time that these disturbances lasted for (bout length) also decreased from both baseline to weeks 5‐10 and baseline to weeks 10‐16; however, this did not reach statistical significance.

### Summary

Table 4 shows the *P* values and effect sizes for the statistically significant well-being parameters. Using G*Power (Heinrich Heine University Düsseldorf) software to deduce the required sample size for a medium effect size (0.5), the optimum number of participants to recruit for a future study would be 34.

**Table 4.** *P* values and effect sizes for statistically significant well-being parameters.

	*P* values	Cohen *d*/Cliffδ**	Summary
**QUALIDEM^a^ (B)^b^**			
Wk 5‐10	.03	0.639	Significant with medium effect
**QUALIDEM (G)^c^**			
Wk 5‐10	.048	0.552	Significant with medium effect
**QUALIDEM (H)^d^**			
Wk 5‐10	.047	0.289**	Significant with high overlap
**Activity (IS)^e^**			
Wk 5‐10	*<*.001	2.601	Significant with large effect
**Activity (IS)**			
Wk 10‐16	*<*.001	1.517	Significant with large effect
**Activity (IV)^f^**			
Wk 5‐10	*<*.001	1.314	Significant with large effect
**Activity (RA)^g^**			
Wk 5‐10	.03	0.621	Significant with medium effect

a QUALIDEM: Quality of Life for People With Dementia.

b B: positive affect.

c G: social isolation.

d H: feeling at home.

e IS: interdaily stability.

f IV: intradaily variation.

g RA: relative amplitude.

## Discussion

### Principal Findings

This study was conducted to determine the effect of dynamic lighting on well-being for people who are living with dementia.

### Social Isolation

Previous studies have highlighted that the exposure to dynamic lighting is not well documented [26-29]. The results from this study highlight that the amount of exposure to dynamic lighting as a group increased from baseline to weeks 5‐10 and 10‐16 by 37.48% and 53.08% respectively. Moreover, the amount of times spent in the common areas increased in comparison to the amount of time spent within their own living quarters. Interestingly, by looking at the QUALIDEM scale for parameter “G” as seen in Figure 10, significant improvements to social isolation were found at weeks 5‐10. This may indicate that exposure to dynamic over static lighting may be responsible for the positive implications found for social isolation. This is in agreement with similar studies conducted by Sust et al [30] who found that residents with dementia experienced improved communications and frequency of participation in activities under dynamic lighting compared to static baseline lighting. Their results were particularly noticed under higher illuminances than delivered in this study (300‐2200 lux), but with dynamic changes to color parallel with those within this study [30].

The reduction in social isolation within our study continued into weeks 10‐16 from baseline but did not reach statistical significance. The reason for the subdued progress may be due to the contrast in lighting within a “circadian synchronization period.” A “circadian synchronization period” refers to the amount of time it takes for the body to adapt to changes in the circadian rhythm; commonly reported as a period of 2‐5 weeks [16]. From baseline to weeks 5‐10, the contrast in lighting transitions is from static lighting to dynamic lighting, whereas there is no contrast introduced in weeks 5‐10 to weeks 10‐16; it is simply a continuance of the dynamic lighting parameters. This dampening in the significant improvement from weeks 5‐10 to nonsignificant improvement in weeks 10‐16 is interesting to consider when comparing with studies by Wong et al [31]. They deciphered that the exposure to previous lighting histories exhibits a huge impact on an individual’s response to lighting [31]. For example, Wong et al [31], found that there is a greater response to lighting interventions upon prior exposure to dark stimuli than bright stimuli, perhaps providing an example of how the larger the contrast in lighting between circadian synchronization periods, the larger the expected impact to the alleviation of certain symptoms. It could be that upon extending our study past 16 weeks, we would have continued to observe a slower paced improvement to social isolation. A further indicator that this might be the case is provided by a study by Figueiro et al [32] who found that symptoms such as agitation, sleep quality, and depression after exposure to dynamic lighting experienced greater alleviation as time progressed in the 25 week study [32].

### Sleep

There is a recurring theme with a lot of our findings in that the most significant improvements are observed from baseline to weeks 5‐10 of dynamic lighting exposure. In most parameter’s cases, this improvement from baseline continues into weeks 10‐16 but at a much lesser and nonsignificant effect than the initial phase. Consider the sleep metrics within this study; the total amount of sleep in hours as highlighted in Figure 14 experiences a progressive increase from baseline to weeks 5‐10 and 10‐16. Again, this improvement follows a steeper incline in the first phase of this study in comparison to the second phase. The aforementioned study by Figueiro et al [32] also demonstrates that between the first 3 weeks and 9 weeks of dynamic lighting exposure there is an improvement from baseline, but with the former interval marginally exceeding the 9 week interval. Since weeks 5‐10 and 10‐16 within our study refer to the first 5 and 11 weeks of dynamic lighting exposure, respectively, our findings would align with the findings from this study [32]. However, it should be noted that there are other studies which have monitored the impact to sleep after exposure to dynamic lighting and found improvements become progressively larger as the exposure interval increases. For example, Hjetland et al [28] found improvements to proxy-rated sleep after 16 and 24 weeks but not after 8 weeks. One reason these results may contrast is that both our study and Figueiro et al [32] placed dynamic lighting in every vicinity that a resident may frequent within a care home, however Hjetland et al [28] solely placed luminaires in the living space. This could suggest that the absence of a positive implication in sleep parameters witnessed by Hjetland et al [28] after 8 weeks of dynamic lighting could be due to the fact that the exposure amounts were not sufficient enough and therefore the impact to the sleep response would take longer to synchronize. Further exploring the sleep data in our study, Figure 14 observes that the number of sleep disturbances reduced from baseline to both phases of the dynamic lighting exposure. Interestingly, it should be noted that participant 5 was the sole resident on sleeping medication and that if this participant’s data are removed from the cohort, the reduction in sleep disturbances at the 10‐16 week dynamic lighting interval becomes significant. This could indicate that people with dementia on sleep medication may require more tailored dynamic lighting to reflect upon their intensified circadian disruption. This is in alignment with studies suggesting that intense sleep disruption leads to a more rapid cognitive decline and more disrupted circadian rhythms acting in a self-depreciating cycle [1].

### Sundowning-Analogous Behavior and Agitation

In terms of sundowning, for most of the participants in our study the percentage of sundowning-analogous activity as seen in Figure 11 demonstrates that after 10‐16 weeks, the majority of participants experience a nonsignificant reduction in sundowning compared to baseline lighting. The sole participants who experienced an increase in this sundowning behavior at this time interval were participants 4 and 7. Interestingly, when calculating the median wake times for residents, these same participants were the latest to wake with times commencing from 8:25 AM and 9:29 AM, respectively. During these times, it is likely that the first lighting these residents will receive is of maximum CS and of blue (peak melanopic) wavelength as seen in Tables2  3, as opposed to a general build up to this level the other residents will have experienced at wake times prior to 8 AM. Considering a study by Colenda et al [33] used bright light therapy for participants immediately as they woke, they found that only 1 of 5 participants experienced positive implications on sleep and agitation measurements [33]. Although a much stronger illuminance was used in their study, it should be considered that timings for peak melanopic lighting immediately upon waking may not be of maximum benefit for well-being.

In addition to this, sundowning seems to be a parameter that progressively improves over time. Again looking at Figure 11, it seems sundowning occurrences seem to increase after a period of 5‐10 weeks before decreasing after a period of 10‐16 weeks. Similar studies by Baandrup and Jennum [34] and Saidane et al [35] have monitored agitation and found no change to agitation after 4 weeks of dynamic lighting and a reduction in the frequency of agitation after 6 months of dynamic lighting intervention [29,34]. This suggests that longer exposure durations may be needed to positively impact this parameter of well-being in dementia. Additionally, a study by Burns et al [36] observed that the reduction in agitation was significantly correlated to the increase in day length, and so may explain why reductions in activity levels during sundowning hours were larger in later weeks as they occurred over the month of June when the summer solstice occurs [36]. In contrast, the QUALIDEM reported agitation scores (parameter D in Figure 10) highlights a nonsignificant increase in agitation from baseline to weeks 5‐10 and 10‐16; however, these results exhibit a nonsignificant decrease in agitation from weeks 5‐10 to 10‐16 of dynamic lighting exposure. Once again, the pattern of larger impacts to symptoms becoming present in the transition from static to dynamic lighting phases compared to marginally extended dynamic lighting phases is apparent. The results of the parameter of agitation are complex to analyze due to the contrasting behaviors in sundowning-analogous and QUALIDEM-reported metrics in our study. Reassuringly, it is commonly reported in the literature that the parameter of agitation in dementia in response to lighting is not well understood. In a 2014 lighting review by Forbes et al [37], they found no significant findings for bright light therapies reducing agitation with one study even contributing to a worsening of symptoms in 5 participants [37,38].

### Positive Affect and Feeling at Home

Our study found significant improvements in both the well-being parameters of “feeling at home” and “positive affect” during weeks 5‐10 of dynamic lighting as seen in Figure 10. Positive affect can be likened to positive mood and similar results after exposure to dynamic lighting have been published by Bromundt et al [39], Figueiro et al [32], Kolberg et al [40], and van Lieushout-van Dal et al [41]. It is interesting to consider whether several parameters within the QUALIDEM scale influence the progression of other parameters; for instance does positive affect influence social isolation or sense of feeling at home? Nevertheless, the positive impact on sense of feeling at home (parameter H in Figure 10) has been replicated in other qualitative studies which have researched the opinion on lighting interventions in dementia [42]. The impact to this parameter of well-being after exposure to dynamic lighting may be less surprising, as is seems unanimous in studies designed to reflect on the environmental opinion of dynamic lighting systems that it is better received than that of static lighting [43,44].

### Rest-Activity

Our study also monitored the changes to rest-activity rhythms upon exposure to dynamic lighting, reporting findings on IS, IV, and RA (see Figures2  11). All three activity metrics experience significant improvements from baseline to weeks 5‐10, with IS reporting significance from baseline to weeks 10‐16 as well. Since these metrics demonstrate adherence of circadian rhythm stability and robustness to light or dark stimuli, it is interesting to consider how the transition to dynamic lighting has impacted them. For example, if we look at Figure 8, which highlights the individual exposures to dynamic lighting, we observe that from weeks 5‐10 to weeks 10‐16 for participants 2, 5, and 8, that they experienced the largest reduction in exposure to dynamic lighting, indicating a suboptimal percentage exposure to dynamic lighting. Consequently, if we look at Figure 10 in the RA plot, we can see that there are spikes at lower values than the trend (green line) for participants 2, 5, and 8. This indicates that insufficient exposure to dynamic lighting can significantly impact the robustness of the circadian rhythm.

Furthermore, these significant improvements in rest-activity upon exposure to dynamic lighting are in agreement with studies undertaken by Arden-van Delft et al [27] who concluded that there may be stabilization in activity after using dynamic lighting over a period of 5.5 months in a recurring control-intervention approach with phases spanning between 28 and 42 days [27]. Other studies such as Baandrup et al [34] found that there was no significant changes to rest-activity after exposure to dynamic lighting over 4 weeks [34]. The reasons for the larger improvement in rest-activity witnessed in our study may be due to the fact the dynamic lighting was provided in a completely ambient fashion even in external common areas. However, it may also be attributed to the fact that rather ironically our activity results are being provided by sensors which are environmentally deployed within their living quarters only (not ambient in common areas), and they are therefore not able to capture rest-activity at times when participants are in the common areas.

### Summary

It seems possible that prolonged use of dynamic lighting for people living with dementia would lead to aggregated improvements to their well-being over time. In this study, the first phase after baseline (weeks 5‐10) experienced a 37.48% increase in dynamic lighting exposure, while the transition from phase 1 (weeks 5‐10) to phase 2 (weeks 10‐16) experienced an additional exposure of 15.6%, which is less than half of the initial static to dynamic transition. Perhaps the lack of statistical significance continued into weeks 10‐16 can be explained by the fact that the dynamic lighting exposure amounts have not increased as much as they did in the first instance; from static (no dynamic exposure) to weeks 5‐10. Since this effect of larger statistical significance is demonstrated in QUALIDEM parameters, rest-activity, and sleep at weeks 5‐10 and not at weeks 10‐16, it seems plausible that insufficient exposure to dynamic lighting could be the cause.

### Study Limitations and Future Work

An overall viewpoint of the technology was communicated by the care home manager who supported its use and benefits. One suggestion was for a central control unit to be introduced whereby the care staff could have control over the light switches in resident’s rooms. This was suggested so that the residents could obtain the majority exposure to the dynamic lighting but may cause issues in implementing due to the fact that it could take the autonomy away from the residents with dementia. The lack of sensors within the light fittings in common areas may have limited the amount of activity data that could be attributed to each individual throughout the day. In future studies, it would be beneficial to request the consent of the use of sensors in these areas and to introduce sensor processing techniques to distinguish between residents within these areas. In addition, the number of participants within this study was small, and undoubtedly a larger cohort (34 participants) would have provided more applicable effect sizes. Implementing a randomized controlled trial to definitively distinguish cause and effect would be the ideal future pathway for research.

Another limitation to this study was that the 16 week trial period took place from March to July which can be considered a “sunny period”; hence, there is the possibility that the changes observed were due to the natural daylight impact. However, the authors carefully considered the 4 week baseline period in order to ensure that the changes that each individual exhibited could always be compared to a baseline for that individual. In this way, even if all months are “sunny periods,” there would be a comparison between static indoor and dynamic indoor lighting. There is a similar argument to be made for the different sun exposures in each room. Since each individual is their own control, these differences are present in both the baseline and intervention measurements and are therefore considered to have minimal impact on any changes observed over the baseline and intervention transition. However as this is a pilot study, future work involves the extension of this study into the winter months to account for external seasonal differences as well.

Additionally, there will be differences in daylight exposure between the equinox in March and the summer solstice in July. The impact of this was not measured but the effect this may have had on study participants cannot be ignored. Again, the authors recommended elongating this study across multiple seasons to gain insight into any potential impact this may administer.

The care home that took part in this study was chosen due to its location within an accessible geographic region for the research team to attend when necessary. Due to the small number of people living with dementia in these facilities, the authors chose to recruit all residents who met the inclusion criteria in favor of random sampling. As a result, the trial was conducted using convenience sampling. The authors note that random sampling may have provided a better representation of the cohort of people living with dementia, but due to the piloting of the trial and the challenges involved in the recruitment of vulnerable adults the authors decided to include all participants. Another limitation was that the type of dementia was not logged. Since several lighting studies have demonstrated that the impact to well-being may be more prominent depending on the type of dementia, this is a parameter that should be logged in all future studies of this type. The authors appreciate that logging this information may provide additional insight into these results.

## Conclusion

A 16-week study trialling dynamic lighting for supporting well-being in a care home for residents with dementia was carried out from March to July 2023. The results found that the use of dynamic lighting over static lighting statistically and significantly improved positive affect, social isolation, sense of feeling at home, and rest-activity within 5 weeks of initial exposure. Nonstatistically significant improvements to sleep amount and disturbances were also recorded and extended even 12 weeks into this study; however, improvements were not observed for the well-being parameter of agitation.

## Abbreviations

CS: circadian stimulus
ipRGC: intrinsically photosensitive retinal ganglion cell
IS: interdaily stability
IV: intradaily variation
M-EDI: Melanopic Equivalent Daylight Illuminance
QUALIDEM: Quality of Life for People With Dementia
RA: relative amplitude
